# Measuring health outcome in young adults with spina bifida

**DOI:** 10.1186/1743-8454-7-S1-S26

**Published:** 2010-12-15

**Authors:** Susana Rodriguez, Esther Pages, Ampar Cuxart, Mar Melendez, Jordi Iborra, Judith Sanchez-Raya

**Affiliations:** 1 Spina Bifida Unit, Rehabilitation Department, Vall d’Hebron Hospital. Barcelona, Spain

## Background

Most young adults with spina bifida (SB) face various impairments and activity limitations and decreased community acceptance which may affect their quality of life and confront young SB patients with additional barriers in the transition from adolescence to adulthood compared to their typically developing peers.

## Materials and methods

We conducted a cross sectional study in 70 adult patients born between 1982 and 1989 with the diagnosis of Myelomeningocele (MMC), Meningocele, Sacral anomalies associated with MMC and Meningocele regularly controlled in our interdisciplinary SB Unit with normal or borderline intelligence quotient (IQ). We collected the data from the medical history and performed an interview with each patient included in this study. The medical data included diagnosis, functional neurological level, shunted hydrocephalus, spinal surgery, faecal and urological reeducation and Hoffer ambulation scale. We developed a questionnaire with 15 secondary conditions associated with SB, which had to be filled in order of importance by all patients. In order to measure their health outcome we administered the EuroQol and the HRQL.

## Results

In relation to EuroQol, patients referred no problems in walking about (35.7%), self-care (72,9%), usual activities (64.1%), pain (64.3%) and anxiety/depression (81.4%). Mean EQ VAS in the EuroQol and HRQL were 66.30 and 196.63 respectively. A higher EQ VAS in the EuroQol was found statistically associated with having boyfriend/girlfriend (p=0.017), and not having some of the conditions of the personal questionnaire such as obesity (p=0.008), urinary infections (p=0.004), and scoliosis (p=0.014). A higher score in the HRQL was associated with a better faecal reeducation (p=0.046), gait level (p=0.043), use of gait aids (p=0.017), family economic level (p=0.020), mobility, self-care, and usual activities. A lower score in the HRQL was related to had undergone spine surgery (p=0.016), having anxiety/depression (p=0.000), and having some of the conditions of the personal questionnaire such us pressure ulcers (p=0.010), and low self-esteem (p=0.024). In the regression model, determinant factors of having worse quality of life measured with EQ VAS were anxiety/depression, obesity, having boyfriend/girlfriend, and urinary infection. Determinant factors of having worse quality of life measured with HRQL were self-care, anxiety/depression, IQ and low self-esteem.

## Conclusions

Almost two thirds of the series referred no problems in the health outcome. The determinants factors related to young SB patients personal perception of quality of life are not those related to disability.

